# Contextual inhibition of fatty acid synthesis by metformin involves glucose-derived acetyl-CoA and cholesterol in pancreatic tumor cells

**DOI:** 10.1007/s11306-013-0555-4

**Published:** 2013-06-26

**Authors:** Mary Jo Cantoria, László G. Boros, Emmanuelle J. Meuillet

**Affiliations:** 1 Department of Nutritional Sciences, The University of Arizona, 1177 East 4th Street, Shantz Building #309, P.O. Box 210038, Tucson, AZ 85721-0038 USA; 2 SiDMAP, LLC, 2990 South Sepulveda Blvd. #300B, Los Angeles, CA 90064 USA; 3 Department of Pediatrics, Los Angeles Biomedical Research Institute at the Harbor-UCLA Medical Center, 1124 West Carson Street, Torrance, CA 90502 USA; 4 The University of Arizona Cancer Center, 1515 N. Campbell Ave Levy Building, Tucson, AZ 85724 USA

**Keywords:** Targeted tracer fate association study, TTFAS, System-wide association study, ^13^C glucose-derived acetyl-CoA, Cholesterol, Contextual drug effect

## Abstract

Metformin, a generic glucose lowering drug, inhibits cancer growth expressly in models that employ high fat/cholesterol intake and/or low glucose availability. Here we use a targeted tracer fate association study (TTFAS) to investigate how cholesterol and metformin administration regulates glucose-derived intermediary metabolism and macromolecule synthesis in pancreatic cancer cells. Wild type K-*ras* BxPC-3 and HOM: GGT(Gly) → TGT(Cys) K12 transformed MIA PaCa-2 adenocarcinoma cells were cultured in the presence of [1,2-^13^C_2_]-d-glucose as the single tracer for 24 h and treated with either 100 μM metformin (MET), 1 mM cholesteryl hemisuccinate (CHS), or the dose matching combination of MET and CHS (CHS–MET). Wild type K-*ras* cells used 11.43 % (SD = ±0.32) of new acetyl-CoA for palmitate synthesis that was derived from glucose, while K-*ras* mutated MIA PaCa-2 cells shuttled less than half as much, 5.47 % [SD = ±0.28 (*P* < 0.01)] of this precursor towards FAS. Cholesterol treatment almost doubled glucose-derived acetyl-CoA enrichment to 9.54 % (SD = ±0.24) and elevated the fraction of new palmitate synthesis by over 2.5-fold in MIA PaCa-2 cells; whereby 100 μM MET treatment resulted in a 28 % inhibitory effect on FAS. Therefore, acetyl-CoA shuttling towards its carboxylase, from thiolase, produces contextual synthetic inhibition by metformin of new palmitate production. Thereby, metformin, mutated K-*ras* and high cholesterol each contributes to limit new fatty acid and potentially cell membrane synthesis, demonstrating a previously unknown mechanism for inhibiting cancer growth during the metabolic syndrome.

**Electronic supplementary material:**

The online version of this article (doi:10.1007/s11306-013-0555-4) contains supplementary material, which is available to authorized users.

## Introduction

Metformin (1,1-dimethylbiguanide) is the first-line oral therapy prescribed for type 2 diabetes (Viollet et al. 2012). It is a potent anti-hyperglycemic and insulin-sensitizing drug that works by decreasing hepatic gluconeogenesis, activating insulin receptor tyrosine phosphorylation (Viollet et al. 2012), decreasing intestinal glucose absorption, and increasing skeletal muscle and adipose tissue glucose uptake (del Barco et al. 2011). Moreover, metformin increases the more active mitochondria-bound hexokinase and actin-bound phosphofructokinase in streptozotocin-induced diabetic male Swiss mice hearts, enhancing glucose sensitivity of those organs (da Silva et al. 2012).

Interestingly, numerous studies have reported a lower risk of cancer (Evans et al. 2005; Monami et al. 2011; Ruiter et al. 2012; Libby et al. 2009) and a reduced risk of cancer-related mortality in diabetics (Bo et al. 2011; Bowker et al. 2006) treated with metformin compared to diabetics that were prescribed other glucose-lowering therapies. Recently, improved survival was observed in diabetic pancreatic cancer patients who were taking metformin (Sadeghi et al. 2012). Published treatment protocols suggest that lactic acidosis is potentially a very serious (Fitzgerald et al. 2009) but a rare side effect of metformin, although the link with metformin has been questioned (Preiss and Sattar 2009).

Various mechanisms of action for metformin’s anti-cancer properties have been published, such as its ability to inhibit the mammalian target of rapamycin complex I (mTORC1) in an AMP activated protein kinase (AMPK)-mediated manner (Mihaylova and Shaw 2011). Other reported mechanisms are the AMPK-independent suppression of mTORC1 activation via inhibition of the Regulator complex (Kalender et al. 2010; Sancak et al. 2008, 2010) and the up regulation of the mTORC1 inhibitor REDD1 (regulated in development and DNA damage responses) (Ben Sahra et al. 2011). Metformin has also been shown to prevent insulin/IGF1 crosstalk with G protein coupled receptor (GPCR) signaling (Kisfalvi et al. 2009) and to induce p53-dependent cell cycle arrest and apoptosis (Ben Sahra et al. 2010b).

Metabolic downstream targets of metformin involve the electron transport chain (ETC) complex I (Whitaker-Menezes et al. 2011; Gonzalez-Barroso et al. 2012; Dykens et al. 2008), which results in energy depletion in cancer cells. The addition of metformin with 2DG induces cell death and promotes ATP depletion, underscoring the importance of oxidative phosphorylation as a cancer therapeutic target (Cheong et al. 2011). In addition, it is demonstrated that metformin inhibits glycolytic flux by suppressing the translocation of glucokinase from the nucleus into cytosol in rat hepatocytes, possibly due to its ATP-depleting properties (Guigas et al. 2006).

In vivo, metformin decreases the expression of acetyl CoA carboxylase, fatty acid synthase and citrate lyase, which are involved in hepatic fatty acid synthesis (Bhalla et al. 2012; Algire et al. 2010). Kim et al. (2011) demonstrated that metformin hinders the AMPK-dependent transactivation of nuclear receptor TR4, which then fails to bind to TR4RE on the *SCD1* 5′ promoter for impairing *SCD1* gene expression. This results in the inhibition of lipogenesis and up regulation of β-oxidation in hepatocytes (Kim et al. 2011).

Metabolic adaptation of transformed mammalian cells to codon K12K-*ras* mutation is identical in fibroblasts (Vizan et al. 2005) and MIA PaCa-2 cells, the latter harboring the GGT → TGT mutation (Lopez-Crapez et al. 1997). The mutant phenotype exhibits greatly increased glycolysis with a low flux along pathways that produce lipid synthesis precursors via the oxidative branch of the pentose cycle, pyruvate dehydrogenase and citrate synthase. The K-*ras* oncogene also mediates a metabolic phenotype that readily trades glucose-derived acetyl-CoA between cholesterol synthesis, controlled by biosynthetic thiolases, and the fatty acid synthase precursor malonyl-CoA, controlled by acetyl-CoA carboxylase. In the presence of either synthetic (C75) or natural (luteolin) FAS inhibitors, cholesterol synthesis readily serves as the alternate route for glucose-derived acetyl-CoA use in MIA PaCa-2 cells (Harris et al. 2012). This channeling of acetyl-CoA between palmitate and cholesterol syntheses serves as the marker of drug efficacies inhibiting metabolic enzymes that compete for the glucose-derived acetyl-CoA substrate.

In the present study we evaluated the metabolic effects of a physiologically relevant dosage of metformin on two pancreatic cancer cell lines. We show metformin, in the context of available acetyl-CoA and cholesterol, limits fatty acid synthesis in pancreatic tumor cells with mutated K-*ras*. This explains how metformin controls K-*ras* induced malignant cell growth via limiting new fatty acid production necessary for cancer cell formation in patients with insulin resistance and the metabolic syndrome. The results of our report provide metabolic explanations for studies showing an anti-cancer effect of metformin in animals fed with a high energy (39.8 % lard) diet (Algire et al. 2008, 2010).

## Materials and methods

### Cell culture and proliferation

BxPC-3 and MIA PaCa-2 pancreatic cancer cells were purchased from American Type Culture Collection (Manassas, VA, USA). Cell culture media, penicillin–streptomycin (P/S) and trypsin–EDTA were purchased from Mediatech (Manassas, VA, USA). BxPC-3 cells were cultured in RPMI media and MIA PaCa-2 cells were grown in DMEM. Both media were supplemented with 10 % FBS from PAA Laboratories, Inc., (Pasching, Austria) and 1 % P/S. The cells were incubated at 37 °C, 5 % CO_2_ and 95 % humidity and passaged with 0.25 % trypsin–EDTA once the cells reached 75–80 % confluence. Cells treated with cholesteryl hemisuccinate (CHS; Sigma-Aldrich, St. Louis, MO), from now on referred to as BxPC3-CHS and MIA PaCa-2-CHS, were incubated in media supplemented with 1 mM CHS complexed to 1 % BSA for 2 weeks prior to metabolomics analysis. The 1 mM cholesteryl hemisuccinate (CHS) dose was used because when compared BxPC-3 (no CHS) versus BxPC-3 (pre-treated with CHS supplementation in the media for 2 weeks) we observed, via western blot, that the CHS-treated cells were more resistant to the AKT inhibitor PH-427, which indicates in vitro biological activity in K-*ras* negative cells.

Cell proliferation was assessed by plating 1 × 10^5^ cells into T-25 cm^2^ flasks. Cells were immediately treated with 100 μM metformin for 72 h as appropriate. The doubling times of BxPC-3 cells and MIA PaCa-2 are 48–60 and 40 h, respectively (Deer et al. 2010). Based on these reported doubling times, we decided to use 72 h for cell proliferation measurements to ensure that the cells have undergone one round of doubling before counting. Cells were then counted using trypan blue exclusion.

#### MTT assay

BxPC-3 and MIA PaCa-2 cells were plated at 2,000 and 500 cells, respectively in 96-well plates and incubated for 24 h in complete RPMI or DMEM media (+1 mM CHS). The following day (day 1), cells were treated with either vehicle (PBS) or 100 μM metformin and incubated for 4 days. On day 5, 50 μL of 3-(4,5-dimethylthiazol-2-yl)-2,5-diphenyltetrazolium bromide (MTT) was added to the wells. After 4 h of incubation, the resulting precipitates were dissolved in 100 μL DMSO. Plates were read at 540 nm using the Synergy 2 Microplate Reader.

### Stable glucose isotope

All reagents were purchased from Sigma-Aldrich (St. Louis, MO) unless otherwise stated. All experiments were conducted in triplicate. Twenty-four hours prior to metformin treatment and metabolomics study, 2 × 10^6^ cells were grown in T-75 cm^2^ culture flasks with glucose and sodium pyruvate-free RPMI and DMEM containing 10 % FBS, 1 % P/S, 4.5 g glucose/l, of which 23–40 % of total final glucose was derived from the [1,2-^13^C]-d-glucose tracer (Isotec, Miamisburg, OH, USA) after media preparation, as measured by GC–MS and reported in Table 1. The tracer was added to the media for all cells along with 100 μM metformin in half of the non-CHS and CHS-treated cells and allowed to incubate for 24 h. Media and trypsinized cell lysates were collected and frozen at −80 °C until analysis.

**Table 1:** Summary of metabolic profiles of BxPC-3 (light shaded columns 3–6) and MIA PaCa-2 (dark shaded columns 7–10) pancreatic adenocarcinoma cells (PDAC)

Metabolite (source-data-matrix-file-log)	Isotopomer fragment dimension	Control	MET	CHS	CHS + MET	Control	MET	CHS	CHS + MET
Glucose content (media-CAS: 50-99-7; **6**)	(mg %; mg/100 mL)	241.33 (±6.75)	254.67 (±3.33)	242.33 (±3.25)	247.67 (±5.01)	247.33 (±6.37)	248.00 (±4.77)	236.67 (±4.04)	240.67 (±3.79)
Glucose consumption (media-CAS: 50-99-7; **7**)	(mg %/hour/million cells)	208.67 (±6.75)	195.33 (±3.33)	207.67 (±3.25)	202.33 (±5.01)	202.67 (±6.37)	202.00 (±4.77)	213.33 (±4.04)	209.33 (±3.79)
Glucose tracer (media-CAS: 138079-87-5; **280**)	^13^C-labeled fraction (*m*/*z*242) (Σm)	23.47 (±0.04)	23.64* (±0.12)	27.87*±(0.04)	28.02** (±0.0002)	30.38^a^ (±0.04)	30.31 (±0.02)	39.32**^, a^ (±0.005)	39.26* (±0.004)
Glucose tracer (media-CAS: 138079-87-5; **283**)	^13^C-m2 (*m*/*z*242) (m2/Σm)	97.80(±0.03)	97.17* (±0.20)	97.49* (±0.12)	97.11**(±0.004)	97.24^a^ (±0.07)	97.14 (±0.04)	96.94**^, a^ (±0.07)	96.93* (±0.12)
Lactate (media-CAS: 50-21-5; **20**)	^13^C-m2 (*m*/*z*328) (m2/Σm)	79.58 (±3.01)	79.86 (±3.06)	81.71 ±(3.15)	77.58 (±3.26)	91.49 (±4.27)	83.64 (±3.37)	80.08 (±3.05)	84.14 (±3.65)
Lactate (media-CAS: 50-21-5; **22B**)	Peak-area (abundance × 10^2^)	4538 (±220)	3174 (±432)	1412** ±(78)	1334** (± 38)	290^a^ ±(20)	2055** (±72)	1827* (±167)	3456* (± 467)
Glutamate (media-CAS: 617-65-2; **78**)	^13^C-m1 (*m*/*z*198) (m1/Σm)	70.67 (±1.3)	71.77 (±1.2)	68.52 (±1.9)	68.34 (±2.1)	47.06^a^ (±0.38)	46.23^a^ (±0.25)	45.64^a^ (±0.75)	43.03*^, a^ (±1.05)
Glutamate (media-CAS: 617-65-2; **79**)	^13^C-m2 (*m*/*z*198) (m2/Σm)	28.65 (±1.15)	27.08 (±0.91)	30.74 (±1.88)	31.17 (±2.17)	38.68^a^ (±1.57)	43.47^a^ (±1.19)	43.57^a^ (±1.78)	46.11*^, a^ (±1.19)
Glutamate (media-CAS: 617-65-2; **81**)	^13^C-m4 (*m*/*z*198) (m4/Σm)	0.29 (±0.03)	0.25 (±0.02)	0.27 (±0.02)	0.32 (±0.01)	10.14^a^ (±0.25)	9.65^a^ (±0.55)	7.62**^, a^ (±0.27)	6.31**^, a^ (±0.12)
Glutamate (media-CAS: 617-65-2; **87B**)	Peak-area (abundance)	150735 (±13440)	173398 (±16925)	168611 (±11332)	87179*^,a^ (±11045)	25566^a^ (±3095)	22591^a^ (±3965)	15879^a^ (±4953)	15861^a^ (±2906)
Palmitate (pellet-CAS: 57-10-3; **98**)	13C-labeled fraction (m/z270) (Σm)	8.95 (±0.22)	8.98 (±0.71)	8.17 (±0.38)	3.86** (±0.22)	4.6^a^ (±0.19)	5.4^a^ (±0.38)	15.3**, ^a^ (±0.48)	11.3**^, a^ (±0.41)
Palmitate (pellet-CAS: 57-10-3; **101**)	Chain elongation-^13^C-m2 (*m*/*z*270) (m2/Σm)	29.78 (±1.60)	29.85 (±0.93)	31.09 (±0.62)	33.27 (±0.30)	39.39^a^ (±0.73)	40.53^a^ (±0.46)	42.41^a^ (±0.98)	40.99^a^ (±0.78)
Palmitate (pellet-CAS: 57-10-3; **102**)	Fraction of new synthesis (FNS) (% of total)	6.23 (±0.04)	6.83 (±0.39)	4.61** (±0.16)	4.94 (±0.39)	6.16 (±0.19)	6.73 (±0.23)	17.16**^, a^ (±0.57)	12.31**^, a^ (±0.61)
Palmitate (pellet-CAS: 57-10-3; **103**)	Ace-CoA enrichment (percent of total)	11.43 (±0.32)	11.06 (±0.36)	11.59 (±1.06)	5.11** (±0.30)	5.47^a^ (±0.28)	6.35^a^ (±0.22)	9.54** (±0.24)	9.43**^, a^ (±0.32)
Cholesterol (pellet-CAS: 57-88-5; **235**)	^13^C labeled fraction (Σm)	17.90 (±0.49)	17.96 (±1.39)	0.06** (±0.003)	0.13** (±0.01)	9.21^a^ (±0.40)	10.80^a^ (±0.76)	0.03**^, a^ (±0.001)	0.04**^, a^ (±0.002)
Cholesterol (pellet-CAS: 57-88-5; **236**)	^13^C content (Σm_n_)	0.57 (±0.06)	0.57 (±0.09)	0.02* (±0.001)	0.01* (±0.0002)	0.23^a^ (±0.03)	0.27 (±0.01)	0.03*^, a^ (± 0.004)	0.02*^, a^ (±0.002)
Cholesterol (pellet- CAS: 57-88-5; **238H**)	Peak-area_CHOL(C:27) (abundance × 10^4^)	3.88 (±0.42)	3.76 (±0.29)	7.32** (±0.30)	7.47* (±0.51)	2.49 (±0.21)	2.42^a^ (±0.27)	5.14*^, a^ (±0.36)	5.32**^, a^ (±0.28)

The metabolic profiles of BxPC-3 and MIA PaCa-2 cells in response to 100 μM metformin after 24 h of culture with and without CHS pretreatment for 2 weeks were obtained via SiDMAP analysis using [1,2-^13^C_2_]-d-glucose tracer and are shown as Ave ± SD

Source-data-matrix-file-log: source of metabolite, i.e.: culture media or pellets with raw data locator file number

M_n_/Σm: Isotopomer/^13^C labeled fraction as SUM(*m*_1_ + *m*_2_ + .. + *m*_n_). Σ*m*_n_:Molar Enrichment (ME) ^13^C content as SUM(1 × *m*_1_ + 2 × *m*_2_ + .. + *n* × *m*_n_) (Lee et al. 1992). Number of observations per group: *n* = 3 (± SD)

*CAS* Chemical abstracts service registry number

* P < 0.05 versus control

** *P* < 0.01 versus control

^a^ *P* < 0.05 versus BxPC-3 (treatment matching comparison between cell lines; where cell lines have been cultured in different media as described in Sect. Methods)

### Product extraction and derivatization

Extraction and derivatization procedures for glucose, cholesterol, fatty acids, lactate, CO_2_ and glutamate were previously published (Harrigan et al. 2006; Harris et al. 2012). Sterols and fatty acids were extracted by saponification of Trizol (500 μL, Invitrogen, Carlsbad, CA) cell extract after removal of the upper glycogen- and RNA-containing supernatant using 30 % KOH and 70 % ethanol (300 μL each) for 2 h. Sterol extraction was performed using 5 mL petroleum ether (EMD, Gibbstown, NJ) with repeated shaking for 20 s three times. The molecular ion of cholesterol was monitored at the *m*/*z* 386 ion cluster. Fatty acids were extracted by further acidification using 6 N hydrochloric acid to pH below 2.0 and repeated vortexing with 5 mL petroleum ether. Fatty acids (palmitate) were monitored at *m*/*z* 270 using canola oil as positive control. The enrichment of acetyl units in media and cell pellet palmitate in response to CHS and metformin treatments was determined using the mass isotopomer distribution analysis (MIDA) approach. Acetyl-CoA and fractions of new synthesis were calculated from the m4/m2 ratio using the formula m4/m2 = (*n*−1)/2·(p/q), where *n* is the number of acetyl units, *p* is the ^13^C labeled precursor acetate fraction and *q* is the ^12^C labeled natural acetate fraction (p + q = 1) (Lee 1996). Additional details of mathematical approaches are described in by Lee et al. (1992) for spectra processing and ^13^C positional distribution diagnostics.

For *glucose extraction*, 500 μL each of 0.3 N barium hydroxide and 0.3 N zinc sulfate were added to 100 μL media. Samples were vortexed and centrifuged for 15 min at 10,000 rpm. Supernatant was dried on air over heat and were derivatized by adding 150 μL hydroxylamine solution and incubated for 2 h at 100 °C followed by addition of 100 μL of acetic anhydride. Samples were incubated at 100 °CC for 1 h and dried under nitrogen over heat as previously described in the fatty acids derivatization section. Ethyl acetate (200 μL) was added. Peak glucose ion was detected at *m*/*z* 187 cluster.

*Lactate* was extracted from media through acidification of 100 μL media with HCl and addition of 1 mL of ethyl acetate. The resulting aqueous layer was dried under nitrogen over heat and derivatized using lactate standard solution as positive control. Two hundred microlitre of 2,2-dimethoxypropane was added followed by 50 μL of 0.5 N methanolic HCl. Samples were incubated at 75 °C for an hour. Sixty microlitre of *n*-propylamine was added and samples were heated for 100 °C for an hour followed by addition of 200 μL dichloromethane. Heptafluorobutyric anhydride (15 μL) was added followed by 150 μL of dichloromethane and samples were subjected to GC/MS. M1 and m2 lactate were differentiated to distinguish the pentose phosphate flux from anaerobic glycolysis (Lee 1996; Lee et al. 1998) and the ion cluster at *m*/*z* 328 was examined.

*Media glutamate* was converted into its *n*-trifluoroacteyl-*n*-butyl derivative and monitored at ion clusters at *m*/*z*152 and *m*/*z*198.

^*13*^ *CO*_*2*_ *Assay* for CO_2_ was generated by adding equal volumes (50 μL) of 0.1N NaHCO3 and 1N HCl to spent media and ^12^CO_2_/^13^CO_2_ ion currents were monitored and calculated from the *m*/*z*44 and *m*/*z*45 peak intensities, respectively, using ^13^CO_2_/^13^CO_2_ of cell culture cabinet’s CO_2_ thank as the reference ratio for ^13^CO_2_ Δ calculations.

### Gas chromatography/mass spectrometry

Agilent 5975 Inert XL Mass Selective Detector connected to HP6890N Network gas chromatograph was used to detect mass spectral data under the following settings: GC inlet 230 °C, MS source 230 °C, MS Quad 150 °C (Harris et al. 2012). For media CO_2_, glucose, lactate and glutamate analyses, an HP-5 column (30 m length × 250 μm diameter × 0.25 μm thickness) was used while a DB-23 column (60 m length, 250 μm diameter × 0.15 μm thickness) was used for fatty acid measurement.

### Statistics

Mass spectral analyses were obtained by consecutive and independent injections of 1 μL sample using an autosampler with optimal split ratios for column loading (10^6^ > abundance > 10^4^ abundance). Data was accepted if the standard sample deviation was below 10 % of the normalized peak intensity (integrated peak area of ion currents; 100 %) among repeated injections. Data download was performed in triplicate manual peak integrations using modified (background subtracted) spectra under the overlapping isotopomer peaks of the total ion chromatogram (TIC) window displayed by the Chemstation (Agilent, Palo Alto, CA) software. A two-tailed independent sample *t* test was used to test for significance (*P* < 0.05, *P* < 0.01) between control and treated groups (*, **) or between cell lines (#).

### Visual system wide association interface

Rapid system-wide association study (SWAS) evaluation of both cell lines was performed by the color assisted visual isotopolome data matrix screening tool (Harrigan et al. 2006), to diagnose phenotypic differences and response to drug treatment.

### Practical note to multiple SWAS entry interpretations

Please note that there is a distinct functional relevance of each value in Table 1, which is the source matrix for the SWAS interface. For example, there are four table entries for palmitate, which show close to equilibrium non-treatment responsive chain elongation of shorter (C14:0) acyl chain by a single acetyl unit from glucose to form ^13^C m_2_ palmitate (101). On the other hand there are significant differences in new palmitate synthesis, which results in altered ^13^C labeled fractions (98), as well as its synthesis from scratch (FNS; 102) with varying glucose derived acetyl-CoA enrichments (103). For System level interpretations we take into account that a significant inhibitory effect of metformin in net new palmitate synthesis from glucose may be considered more rate limiting on new membrane synthesis and cell proliferation, while its effect on elongating a previously existing shorter acyl chain is not affected. Therefore, multiple SWAS interface entries for the same product clarify the potential biological impact(s) of MET treatment on important precursor-product relationships in a complex biological system.

## Results

### Cell viability

**Fig. 1:**
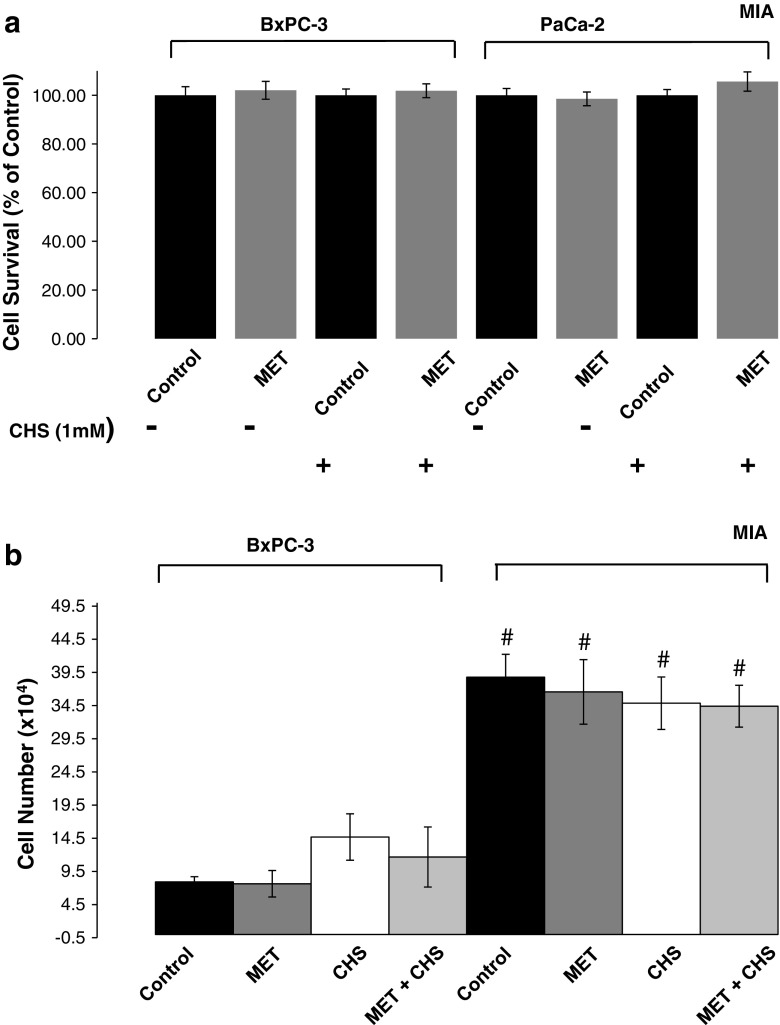
**a** Cell survival of various pancreatic adenocarcinoma cell lines treated with metformin. MTT (3-(4,5-dimethylthiazol-2-yl)-2,5-diphenyltetrazolium bromide) assay was performed to measure cell viability in BxPC-3 and MIA PaCa-2 cells after treatment with metformin (100 μM, MET) in the absence or presence of cholesteryl hemisuccinate (CHS) pre-treatment for 2 weeks. *Dark bars* are control and light bars are MET-treated cells. All data are mean ± SD (*n* = 3 per group). **b** Cell proliferation of various pancreatic adenocarcinoma cell lines treated with metformin. Cell proliferation was assessed by plating 1 × 10^5^ cells into T-25 cm^2^ flasks in triplicate. Cells were immediately treated with 100 μM metformin for 72 h as appropriate. Cells were then counted using trypan blue exclusion. We used a relatively short (72 h) incubation time for MET treatment, which showed a slowing trend in MIA proliferation with no (yet) significant differences but decreasing NS *P* values [Fig. 1b; (*P* = 0.293-MET; 0.139-CHS; 0.089-CHS + MET)]. BxPC-3 cells fell short of showing initial response to MET (*P* = 0.425-MET; 0.118-CHS; 0.127-CHS + MET)

The ability of metformin (MET, 100 μM) to affect cell viability of various PDAC cell lines with and without CHS pre-treatment for 2 weeks was examined using MTT assay (Fig. 1a). Metformin alone was unable to decrease cancer cell viability after 4 days of drug treatment. Hence, the metabolic impacts of CHS and metformin in this study cannot be attributed to cell death inducing properties.

### Cell proliferation

The ability of MET to affect cell proliferation for 72 h in all groups was assessed by counting using the trypan blue exclusion method. MET treatment did not significantly alter cell proliferation in control or CHS-treated cells (Fig. 1b). As expected, MIA PaCa-2 cells showed shorter doubling times than BxPC-3 cells did.

### Heavy [1,2-^13^C_2_]-d-glucose enrichment and cholesteryl hemisuccinate (CHS) media preparation

**Fig. 2:**
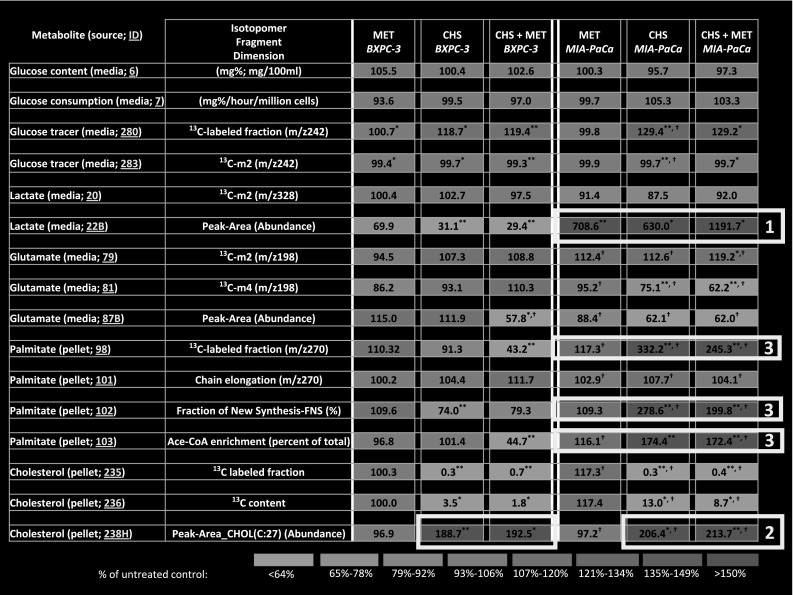
EZTopolome(K-ras); isotopolome-wide association study (IWAS) array showing heat map [percent changes to untreated control (100 %)] of flux responses associated with CHS and MET treatment in BxPC-3 and the mutant K-ras (MIA PaCa-2) PDAC cell lines. EZTopolome(K-ras) contains group averages from Table 1 as percent of control values in an identical, coherent matrix format [please note control 100 % values are omitted for EZTopolome (K-ras)]. Visual system-wide association study (SWAS) evaluations show the significant phenotypic differences as well as effects of CHS and MET for a rapid overview of Results. *P < 0.05 versus control; **P < 0.01 versus control; †P < 0.05 versus BxPC-3 (treatment matching comparison between cell lines)

There is a uniform decrease in ^13^C-glucose labeled fraction in the media with identical tracer carbon substitutions [1,2-^13^C_2_]-d-glucose of non-CHS-treated BxPC-3 and MIA PaCa-2 cells in comparison with their cell-specific controls (Fig. 2—EZTopolome (K-ras) ID 280 and 283; Table 1, media ^13^C glucose panel 280 and 283). This difference is consistent with the increased natural ^13^C labeled glucose ratio of the excess media that was replaced with CHS in bovine albumin for CHS treatment. Instead of providing calculated values, we report, as determined by GC–MS, exact [1,2-^13^C_2_]-d-glucose enrichment after preparing all FBS, albumin and CHS supplemented DMEM and RPMI in Table 1. More specifically, there were a relative 18.8 (±0.15) and 29.4 % (±0.01 %) differences in CHS solution treated RPMI (BxPC-3) and DMEM (MIA PaCa-2) in [1,2-^13^C_2_]-d-glucose enrichment (please note that glucose consumption between cell lines and among treatments remains unaffected), which are shown in Fig. 2—EZTopolome(K-ras) ID 6 and 7; Table 1, media glucose panel 6 and 7, before and after the 24-h culturing period. Due to the expected and observed differences in [1,2-^13^C_2_]-d-glucose in the CHS containing media, below we report either ^13^C isotope ratios in glucose-derived isotopomer products as positional ^13^C enrichment (*m*_n_/*m*_k_) or divide isotopomer extracted ion chromatogram (EIC) by the ^13^C labeled fraction (m_n_/Σ*m*). These isotopomer markers of glucose to product flux show [1,2-^13^C_2_]-d-glucose tracer distribution and thus readily reflect changes in cells’ phenotypes after MET treatment. In other words, normalized isotopomer distribution patterns are independent of the amount of tracer uptake, while product concentrations are reported as total ion currents that include unlabeled and labeled fractions, alike. In simple words m_n_/Σ*m* reflects how cells use a single glucose molecule as surrogate markers of flux. This is consistent with the use of [1,2-^13^C_2_]-d-glucose as a true tracer for investigating metformin’s effect on cultured tumor cell metabolism and its branching routes. To this end, for example, the identical ~97 % media glucose labeled specifically on the 1,2 carbon positions of the ^13^C glucose fraction (*m*_2_/Σ*m*) indicates that there was truly negligible glucose release by cultured cells via gluconeogenesis, necrosis and glucose production to scramble the glucose tracer (Fig. 2—EZTopolome(K-ras) ID 283; Table 1, media ^13^C glucose panel 283; please note the small SD values characteristic of the *m*_*n*_/Σ*m* mathematics in isotopomer analysis methods).

### Complete glucose oxidation

**Fig. 3:**
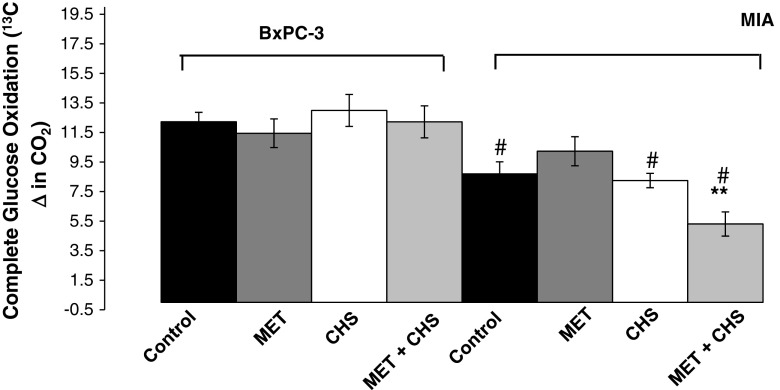
Complete glucose oxidation of BxPC-3 and MIA PaCa-2 pancreatic adenocarcinoma cells in response to 100 μM metformin after 24 h of culture with and without CHS pretreatment for 2 weeks. Treatment with a combination of CHS and metformin in MIA PaCa-2 cells showed a significant inhibition of the TCA cycle measured by a decrease in glucose oxidation. Control = cells grown in media, MET = cells treated with metformin (100 μM) for 24 h, CHS = cells pre-treated with 1 mM CHS for 2 weeks, CHS + MET = cells pre-incubated with 1 mM CHS for 2 weeks then treated with metformin (100 μM) for 24 h. All data are mean ± SD (*n* = 3 per group). **P < 0.01; ^#^ *P* < 0.05 between cell lines

The decrease in *complete glucose oxidation* (Fig. 3) into ^13^CO_2_ observed in the MIA PaCa-2 cells after the combined CHS and MET treatment indicates that metformin decreases direct and indirect glucose oxidation relative to that of amino- and fatty acids (unlabeled substrates) for ATP synthesis. Thus, K-*ras*-mutated MIA PaCa-2 cells, pre-treated with CHS, respond with a decrease in TCA cycle glucose-derived oxaloacetate and citrate turnover, anaplerosis and oxidation.

### Lactate synthesis

We observed an expected over 75 % 13C m2 lactate via glycolysis in the glucose derived (labeled) lactate species in media (Fig. 2—EZTopolome(K-ras) ID 20 and 22B; Table 1, media lactate panel 22 and 22B). On the other hand, ^13^C m_2_ glutamate positional labeling, which is a surrogate of pyruvate dehydrogenase activity for pyruvate’s entry into the TCA cycle, increased in CHS-MET MIA PaCa-2 cells, supporting metformin’s ability to increase TCA cycle cataplerosis at the expense of anaplerosis (anabolic use of pyruvate for new net oxaloacetate and citrate production, also confirmed with increasing m2/m1) in this group (Fig. 2—EZTopolome(K-ras) ID 79 and 81; Table 1, media glutamate panel 79 and 81). Extracellular glutamate concentration TIC surrogates shown as GC/MS peak areas decreased in both cell lines after CHS and MET treatments, which also indicates a uniform decrease in ketoglutarate and glutamate output of TCA cycle (Fig. 2—EZTopolome(K-ras) ID 87B; Table 1, media glutamate panel 87B). While glutamate’s ^13^C m_4_ fractions are small in wild type K-*ras* BxPC-3 cells (<1 %), there is a prominent ^13^C m_4_ glutamate fraction in K-*ras* mutated MIA PaCa-2 cells (Table 1, media glutamate panel 81). In MIA cells CHS and CHS + MET prominently inhibits oxaloacetate’s replenishment from glucose for new citrate synthesis via pyruvate carboxylase and by repeated cycling. Due to decreased m1 (Table 1, 78) pyruvate carboxylase is also a potential target of the CHS + MET treatment.

### Fatty acid palmitate synthesis

Significant phenotypic differences between BxPC-3 and MIA PaCa-2 cells continue in terms of de novo *fatty acid synthesis* deriving from the tracer glucose. There is an 8.95 % (±0.24 %) of glucose-derived palmitate labeled in BxPC-3 cells, while only 4.61 % (±0.20 %) (~half) in MIAPaCa-2 (Table 1, pellet palmitate panel 98). This shows that at baseline, MIA PaCa-2 cells are less lipogenic from glucose in comparison with control BxPC-3. Both cell types reach equilibrium in palmitate’s acetyl-CoA enrichment from glucose after 4 h of culturing (data not shown).

### Sterol ring synthesis

As cholesterol and de novo fatty acid syntheses compete for acetyl-CoA, external cholesterol (CHS) administration blocked new sterol synthesis shown by the severely decreased ^13^C labeled cholesterol fractions with severely increased concentrations (total ion current) values (Table 1, pellet cholesterol panel 235, 236, 238H). However, in K-*ras* transformed cells the addition of cholesterol in the form of CHS increased the glucose derived acetyl-CoA enrichment and the fraction of newly synthesized (FNS) palmitate from the tracer glucose derived acetyl-CoA. Cholesterol supplementation had no effect on BxPC-3′s already high glucose-derived acetyl-CoA enrichment in palmitate. Hence, addition of CHS did not increase de novo palmitate synthesis in BxPC-3 cells, yet, there was an up-regulation, close to double, in glucose-derived synthesis of new palmitate in CHS-supplemented MIA PaCa-2 cells (Fig. 2—EZTopolome(K-ras) ID 102, 103; Table 1, pellet palmitate panel 102, 103). CHS + MET treatment significantly decreased de novo palmitate synthesis both BxPC-3 versus control and MIA PaCa-2 versus CHS. This suggests that metformin clearly is able to inhibit glucose-derived acetyl-CoA flux via fatty acid synthase in the context of acetyl-CoA availability and its consumption by acetyl-CoA carboxylase when sterol synthesis is blocked.

### System wide associations

The rapid system-wide association study (SWAS) evaluation of both cell lines, using the color assisted visual isotopolome data matrix screening tool (Harrigan et al. 2006), confirmed phenotypic differences by increased lactate production in treated MIA PaCa-2 cells [Fig. 2—EZTopolome(K-*ras*) media 22B; square labeled as 1], the ready uptake of cholesteryl-hemi succinate by both cell lines [Fig. 2—EZTopolome(K-*ras*) pellets 238H; squares labeled as 2], acetyl-CoA shuttling towards newly synthesized palmitate [Fig. 2—EZTopolome(K-ras) pellets 102 and 103; squares labeled as 3] in the presence of CHS.

**Fig. 4:**
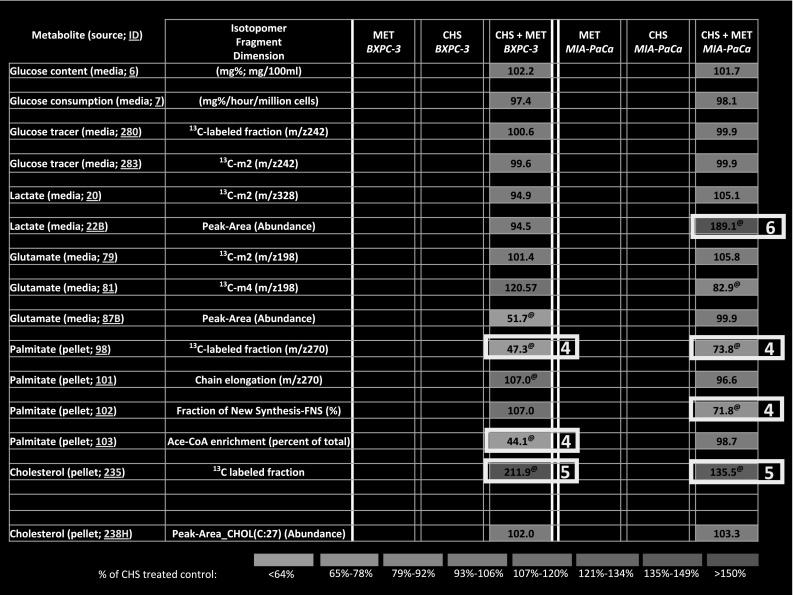
EZTopolome(CHS-MET); isotopolome-wide association study (IWAS) array showing heat map [percent changes to CHS treated control (100 %)] of flux responses associated with MET treatment in BxPC-3 and the mutant K-ras (MIA PaCa-2) PDAC cell lines. EZTopolome(CHS-MET) contains group averages from Table 1 as percent of CHS values in an identical, coherent matrix format [please note CHS 100 % values are omitted for EZTopolome(CHS-MET)]. Visual system-wide association study (SWAS) evaluations show significant phenotypic differences after CHS treatment, as well as effects of MET for a rapid overview of Results. (@, *P* < 0.05 in comparison with CHS treated control (100 %); cholesterol ^13^C content 236 is not shown for comparison due to low values after external CHS treatment)

On the other hand, rapid system-wide association study (SWAS) evaluation of Metformin effect in addition to CHS treatment (100 %) showed a significant decrease in newly synthesized palmitate fraction via FAS (m4/m2) [Fig. 4—EZTopolome(CHS-MET) media 102; square labeled as 4], the re-labeling of cholesterol in both cell lines [Fig. 4—EZTopolome(CHS-MET) pellets 235; squares labeled as 5], consistent with less acetyl-CoA used for palmitate synthesis, as well as further lactate disposal from glucose in the K-*ras* positive cells (Fig. 4—EZTopolome(CHS-MET) pellets 235; squares labeled as 6) in the presence of CHS.

## Discussions

Various studies have implicated metformin as a potential anti-cancer agent. However, metformin’s mechanism of action against cancer remains to be determined (Pollak 2012). Because metformin affects critical metabolic pathways to ameliorate diabetic symptoms, and because cancer cell proliferation is dependent upon altered metabolism, we investigated how this drug controls metabolic flux in two PDAC cell lines, BxPC-3 and MIA PaCa-2, using [1,2-^13^C_2_]-d-glucose as the tracer and GC/MS. We used the stable isotope-labeled dynamic metabolic profiling (SiDMAP) (Boros et al. 2003) approach as ^13^C tracers provide the most comprehensive means of characterizing cellular metabolism and uniquely labeled ^13^C substrates offer probes of specific reactions within complex networks. The choice of tracer largely determines the precision available to estimate metabolic fluxes in complex mammalian systems, with [1,2-^13^C_2_]-d-glucose providing the most precise estimates for glycolysis, the pentose phosphate pathway, and the overall metabolic network (Metallo et al. 2009).

**Fig. 5:**
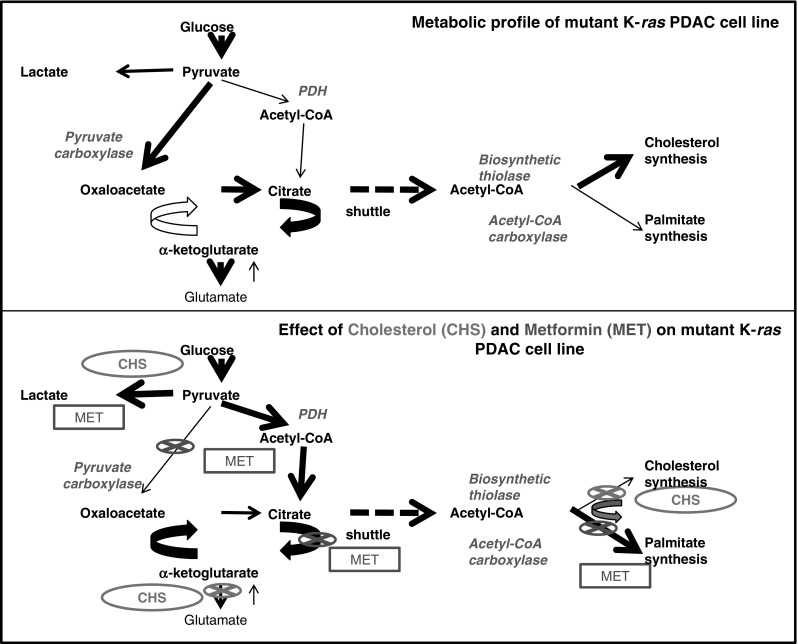
Metabolic profile changes associated with CHS and MET treatment in mutant K-*ras* (MIA PaCa-2) PDAC cell lines. At baseline, the mutant K-*ras* cancer cells exhibit less efficient glucose oxidation and low fatty acid synthase flux with cholesterol readily synthesized. CHS treatment (*green*) blocks cholesterol synthesis, by which glucose-deriving acetyl-CoA is diverted towards fatty acid synthase, instead of new cholesterol synthesis. This is when addition of metformin (*red*) gains a functional fatty acid synthase inhibitory effect. This demonstrates the contextual System effects of mutated K-*ras*, cholesterol and metformin in the metabolic syndrome to inhibit potentially membrane production and cancer growth. Please note that hypotheses for further testing are suggested as (1) the effect of CHS on glut-aminotransferase, (2) further evidence for MET inhibition of the citrate arm of the TCA cycle and (3) pyruvate carboxylase, which is only significant in the presence of CHS

In this study, there may be indication that metformin controls PDAC cell metabolism by inhibiting TCA cycle anaplerosis and de novo fatty acid palmitate synthesis from glucose-derived acetyl-CoA. For an overview, please see Fig. 5. These effects were only observed in MIA PaCa-2 cells that were pre-treated with 1 mM CHS for 2 weeks. Although previous studies (Meuillet et al. 1999a, b) implicated that cholesterol supplementation causes a reduction in plasma membrane fluidity, we herein show that cholesterol also alters cellular metabolism by redirecting glucose-derived acetyl-CoA towards fatty acid palmitate synthesis, a change through which metformin gains its contextual efficacy to inhibit FAS, an important target to control cancer cell proliferation (Little and Kridel 2008; Menendez and Lupu 2007). Metformin may also control pancreatic cancer cell growth in diabetes and obesity by limited TCA cycle anaplerosis, an observation that provides a hypothesis for further testing.

In dose escalating studies 1 mM metformin has been reported to potentiate the cell proliferation inhibitory effect of the hexokinase inhibitor 2DG (Sandulache et al. 2011). At a higher concentration (5 mM), metformin was shown to cause cell death when combined with 2DG (Cheong et al. 2011). In the present study, we show that a physiologically relevant dosage of metformin (100 μM) (Wiernsperger and Rapin 1995) is able to impair glucose utilization through inhibition of FAS when new cholesterol synthesis is limited. We raise for the first time that metformin may inhibit pyruvate carboxylase flux, indicated by decreased m_1_ but increased m_2_ in glutamate, TCA cycle output and likely ATP production (not measured) in the CHS-MIA PaCa-2 cancer cell line. In support of the role of metformin in ATP depletion, others have published evidence indicating that metformin only and when combined with 2DG decreases total ATP in human gastric cancer parenteral p-SK4 (Cheong et al. 2011) and prostate cancer cells LNCaP (Ben Sahra et al. 2010a, b), compared to their untreated controls. Previous studies have also implicated contextual factors that enable metformin’s anti-cancer properties (Menendez et al. 2012).

Palmitate is the sole product of FAS and its dependence on acetyl- and malonyl-CoA availabilities is evident; palmitate’s 13C positional labeling from glucose-derived acetate demonstrates a robust, over twofold increase in response to CHS. As cellular metabolic reprogramming is evident after cholesterol pre-treatment in pancreatic cancer cells, the same may occur in the obese diabetic cancer patient with increased circulating cholesterol. The presence of cholesterol establishes the flux-based context in which efficacies of metformin are high because of tissue specificities in which FAS gene expression is already high due to negative feedback (low product concentrations). Such modalities include pancreatic cancer (Walter et al. 2009).

Interestingly, in primary cultured rat hepatocytes, metformin affected neither fatty acid oxidation nor triglyceride synthesis (Fulgencio et al. 2001), yet in an in vivo model of colon (Algire et al. 2010) and hepatocellular carcinoma (HCC) (Bhalla et al. 2012) with circulating cholesterol, metformin readily decreased FAS expression. In our study metformin was effective in altering palmitate synthesis only after glucose-derived acetyl-CoA was re-directed towards acetyl-CoA carboxylase from biosynthetic thiolase, HMG-CoA and cholesterol synthesis by CHS administration. This finding suggests that metformin may inhibit acetyl-CoA carboxylase, which has been suggested as a cancer promoting enzyme (Wakil and Abu-Elheiga 2009), providing malonyl-CoA precursor directly for FAS.

Determining the cause of the apparent differences in the effects of metformin between BxPC-3 and MIA PaCa-2 cell lines represents an exciting research endeavor. A recent study has shown that, in vitro, RAS diffusion is slowed after cholesterol loading in COS-7 cells (Goodwin et al. 2005). Given the evidence that mutations in K-*ras* show distinct metabolic phenotypes (Vizan et al. 2005), it is possible that difference in K-*ras* status between BxPC-3 (WT K-*ras*) and MIA PaCa-2 (mutated K-*ras*), besides apparent differences in the culture media, contribute significantly to their diverse response to cholesterol, with MIA PaCa-2 being responsive by increasing acetyl-CoA availability for FAS, comparable to that of BxPC-3. After this metabolic adaptation of MIA PaCa-2 cells to glucose-derived acetyl-CoA shuttling towards FAS, metformin acts as an inhibitor of new fatty acid synthesis, while in BxPC-3 metformin dilutes glucose-derived acetate with no apparent decrease in the rate of new palmitate formation via FAS. Despite the numerous genetic and phenotypic differences between BxPC-3 and MIA PaCa-2 cells (Deer et al. 2010), it is evident that extracellular cholesterol uniformly decreases ^13^C labeling for intracellular cholesterol synthesis in both cell lines. Consequently, extracellular cholesterol increases acetyl-CoA shuttling towards FAS from glucose in MIA PaCa-2 cells. The sterol ring is an unrecyclable carbon sink when newly synthesized from glucose derived acetyl-CoA in cells; therefore CHS as an external supply introduces significant effects in redistributing acetyl-CoA among cholesterol and fatty acid synthesis pathways, as shown in our paper. This necessitates the introduction of ^13^C tracer-based metabolic flux research tools in the genetic and signaling research agendas of human cancers as well as metabolic diseases in order to better understand the response of whole biological systems to common drugs.

We acknowledge a potential limitation of this study, succinate of CHS being a potential substrate for TCA cycle metabolism. The dose at which CHS was administrated (1 mM) is 1/25th of that of glucose (4.5 g/glucose/L (25mM)) in media. We observed no significant decrease in 13CO2Δ values after CHS treatment, which is an important assurance that this hemisuccinate did not dilute the TCA cycle substrate pool to any measurable extent. No such dilution was expected from cholesterol under any circumstance due to its stable C:27 carbon ring that lacks oxidation by mammalian cells.

Another limitation may be that this study did not test cell membrane synthesis/turnover directly from isolated membranes for their labeled palmitate pool. We use the connection between inhibited FAS and limited cell membrane synthesis because undifferentiated cells contain the majority, over 90 %, of phospho-sphingo- and triglyceride-derived fatty acids in nuclear and plasma membranes. This fraction yields most derivatized methyl-palmitate for GC–MS analyses after saponification of tumor cell pellets. Previous work with fractionated fat pools of cultured undifferentiated murine myoblasts (Espinoza et al. 2010) confirms the assumption that transformed cell use FAS for new membrane synthesis and proliferation. Palmitate synthesis via FAS for new membrane formation became a target to treat cancer (Flavin et al. 2010 for review). A similar mechanism is suggested herein for metformin in the presence of cholesterol.

Whilst the four measured metabolites and their ^13^C isotopomer ratios from glucose generate a highly informative matrix, they do not describe the full extent of glucose metabolism. Published methods are available for isotopolome‐wide labeling studies with LC‐MS (Creek et al. 2012) and GC‐MS (Hiller et al. 2013). Targeted tracer fate association studies (TTFA or TTFAS) after drug treatment may provide significantly more information in the future than do either a non-targeted tracer fate detection (NTFD) approach or a limited product IWAS. It is important to point out that even a relatively low but steady increase in the rate of glucose-derived new acetate can contribute to enlarged palmitate pools, over time. Even though there are only a few percent increases in glucose-derived acetyl-CoA to new palmitate synthesis above that in control cells, this surrogate marker of newly contributed acetyl-CoA yields a potentially large new palmitate pool for membrane synthesis; although the majority, ~85 % of acetyl-CoA are still recycled from existing (unlabeled) fatty acids, similar to other transformed cell systems (Bulotta et al. 2003). Another important point is that glucose is a reliable source for new acetyl-CoA synthesis as plasma concentrations, especially in diabetes, are constantly high. In the metabolic syndrome this is combined with high circulating cholesterol, which together yields a reliable new acetyl-CoA pool (glucose) and an inhibitor of new cholesterol synthesis (cholesterol) for tumor cells to thrive with more new palmitate. Metformin limits this new fraction of palmitate synthesis in the context of metabolic changes in a diabetic host, potentially, based on our observations.

Using the same principles as genome-wide association studies (GWAS), this paper demonstrates the effect of metformin by a targeted isotopolome-wide association study (IWAS) approach. This is readily expanded towards system-wide associations (SWAS) when comparing specific metabolic fingerprints, as well as the effect of Metformin in the presence of nutritional factor cholesterol in obesity, in two genetically diverse tumor cell lines. Although it may seem ambitious, IWAS presented in a heat map (EZotopolome) reveals that metformin under high cholesterol contributes to limit new fatty acid and potentially plasma and nuclear membrane synthesis, demonstrating a previously unknown mechanism for inhibiting cancer growth during the metabolic syndrome.

## Concluding remarks

In conclusion, metformin possesses FAS inhibitory properties in the context of the combined metabolic effects of available acetyl-CoA and extracellular cholesterol. Such contextual synthetic inhibition of FAS by metformin may partly explain the drug’s demonstrated ability to decelerate growth in some cancers of the diabetic patient (Li et al. 2009) or patients with metabolic syndrome. One of the observed side effects, lactic acidosis, is also consistent with our report that the product of glucose metabolism is lactic acid when cholesterol and fatty acid new syntheses are inhibited in the presence of MET.

## Electronic supplementary material

Below is the link to the electronic supplementary material.

Supplementary material 1 (PDF 134 kb)

## Abbreviations

HMG-CoA: 3-Hydroxy-3-methylglutaryl-CoA
FAS: Fatty acid synthase
SIDMAP: Stable isotope-based dynamic metabolic profiling
HOM: Homozygous
MET: Metformin
CHS: Cholesteryl hemisuccinate
K-*ras*: Kirsten rat sarcoma viral oncogene homolog
Luteolin: 2-(3,4-Dihydroxyphenyl)-5,7-dihydroxy-4-chromenone
PDAC: Pancreatic ductal adenocarcinoma
GC/MS: Gas chromatography–mass spectrometry
HCC: Hepatocellular carcinoma
G6PDH: Glucose-6-phosphate dehydrogenase
IWAS: Isotopolome-wide association study
SWAS: System-wide association study
*EZTopolome* (reference): Isotopolome-wide association study array (normalized)
TTFA: Targeted tracer fate associations
TTFAS: Targeted tracer fate association study
