# Supplementary material for: Contextual inhibition of fatty acid synthesis by metformin involves glucose-derived acetyl-CoA and cholesterol in pancreatic tumor cells
Source: Metabolomics. 2013 Jun 26;10(1):91–104. doi: 10.1007/s11306-013-0555-4 (PMC3890070; doi:10.1007/s11306-013-0555-4)
Supplement: Supplementary file 1 — Supplementary material 1 (PDF 134 kb) [file 11306_2013_555_MOESM1_ESM.pdf]

Supplemental Material

Supplemental Figure 1 - Intracellular tracer-derived acetate enrichment for fatty acid synthesis of BxPC-3 and MIA PaCa-2 pancreatic adenocarcinoma cells in response to 100 µM metformin after 24 h of culture with and without CHS pretreatment for two weeks. BxPC-3 cells treated with CHS and metformin show inhibition of acetate enrichment for palmitate (the only product of fatty acid synthase) synthesis indicating inhibition of FAS. MIA PaCa-2 cells treated with CHS only and a combination of CHS and metformin show increased acetate enrichment for *de novo* palmitate synthesis as a consequence of fatty acid futile cycling. All data are means ± SD (n = 3 per group). **, $P < 0.01$, # $P < 0.05$ between cell lines. See Fig. 2 for x-axis labeling

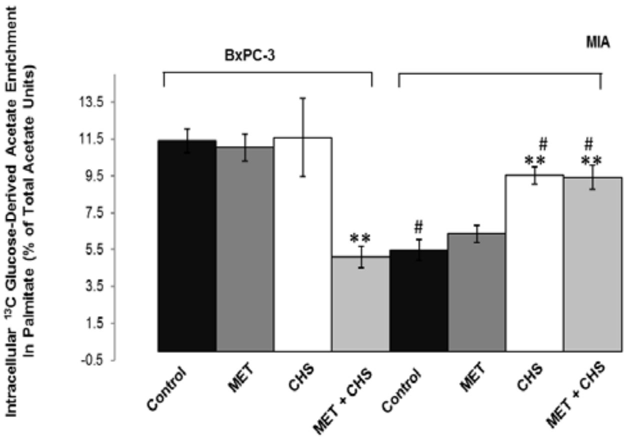

Supplemental Figure. 1

• DOI: 10.1007/s11306-013-0555-4

Supplemental Figure 2 - Intracellular palmitate turnover via direct synthesis from tracer-derived acetate of BxPC-3 and MIA PaCa-2 pancreatic adenocarcinoma cells in response to 100 μM metformin after 24 h of culture with and without CHS pretreatment for two weeks. No significant difference is observed between the two cell lines in terms of the rate of baseline glucose-derived *de novo* fatty acid synthesis. MIA PaCa-2 cells show a significant increase in the rate of *de novo* fatty acid synthesis from the glucose tracer after CHS pre-treatment indicating a shift from cholesterol synthesis to fatty acid metabolism due to CHS supplementation. MET treatment significantly antagonizes this CHS effect to decrease fatty acid synthesis rate. **, $P < 0.01$, # $P < 0.05$ between cell lines. See Fig. 2 for x-axis labeling

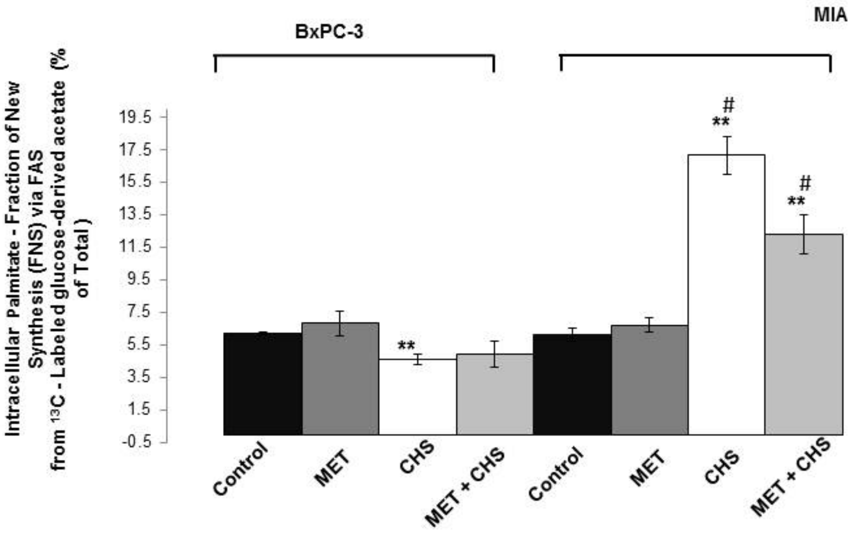

Supplemental Figure. 2

• DOI: 10.1007/s11306-013-0555-4
